# *Rhizophagus irregularis* improves Hg tolerance of *Medicago truncatula* by upregulating the Zn transporter genes *ZIP2* and *ZIP6*

**DOI:** 10.1007/s00572-022-01100-6

**Published:** 2023-01-10

**Authors:** Yaqin Guo, Nadine Sommer, Konrad Martin, Frank Rasche

**Affiliations:** 1 Department of Agronomy in the Tropics and Subtropics, Institute of Agricultural Sciences in the Tropics (Hans-Ruthenberg-Institute), University of Hohenheim, 70593 Stuttgart, Germany; 2 Department of Crop Physiology of Specialty Crops, Institute of Crop Science, University of Hohenheim, 70593 Stuttgart, Germany

**Keywords:** Heavy metal (HM), Arbuscular mycorrhizal fungi (AMF), Hg uptake, Hg accumulation, Zn transporters

## Abstract

Mercury (Hg) pollution of soils is a critical environmental problem. To rehabilitate Hg contaminated soils, arbuscular mycorrhizal (AM) fungi-based phytoremediation may be supportive, yet the functional potential of AM fungi in response to Hg exposure is unclear. In a greenhouse experiment, we assessed the response of *Medicago truncatula* (Hg tolerance index (TI), Hg partitioning) to different Hg concentrations [0 (Hg0), 25 (Hg25), 50 (Hg50) µg g^−1^] in treatments with (AM) and without (NM) inoculation of *Rhizophagus irregularis*. Additionally, zinc (Zn) uptake and the expression of two Zn transporter genes (*ZIP2*, *ZIP6*) were examined because Zn is an essential element for plants and shares the same outer electronic configuration as Hg, implying potential competition for the same transporters. The results showed that AM plants had a higher TI than NM plants. Plant roots were identified as dominant Hg reservoirs. AM inoculation reduced the root Hg concentration under Hg50 compared to the NM treatment. There was an interaction between Hg treatment and AM inoculation on Hg stem concentration, i.e., at Hg25, AM inoculation decreased the Hg translocation from roots to stems, while Hg translocation was increased at Hg50 compared to the NM treatment. Zn acquisition was improved by *R. irregularis*. The negative relationship between Hg and Zn concentrations in the roots of AM and NM plants implied potential competition for the same transporters, although the expression of Zn transporters was upregulated by AM inoculation at all Hg levels. In conclusion, this baseline study demonstrated that *R. irregularis* may play an important role in Hg tolerance of *M. truncatula*, suggesting its potential for Hg-contaminated phytoremediation.

**Supplementary Information:**

The online version contains supplementary material available at 10.1007/s00572-022-01100-6.

## Introduction

Mercury (Hg) is a very toxic heavy metal (HM), ranked as the third most hazardous substance on earth (ATSDR 2019). Because of its high mobility and persistence in the environment, Hg has been recognized as a global pollutant and major threat to human health (Driscoll et al. 2013). Pollution with Hg occurs naturally by weathering and to a large extent through anthropogenic activities. These include artisanal gold mining, stationary combustion of coal, nonferrous metals production, and cement production. Among these, artisanal gold mining is the world’s largest source of anthropogenic Hg emission (EPA 2018) and is done in many regions around the world (Wang et al. 2022). This practice often is uncontrolled and aggravates the distribution of Hg in terrestrial ecosystems, which may affect the growth of food crops, thereby compromising food safety (Gworek et al. 2020).

Decontamination of Hg polluted soils is a major concern in environmental legislation and food production. Phytoremediation, a plant-based technology, offers a green and sustainable solution to remediate Hg contaminated soils. Phytoextraction (facilitating Hg translocation to plant stems) and phytostablization (facilitating Hg storage in plant roots, while preventing Hg translocation to plant stems) are two popular approaches of phytoremediation of soil Hg contamination (Bhat et al. 2022). Plants, however, may suffer from negative effects of Hg toxicity, which limits plant growth and even threatens plant survival. To counteract such negative impacts on plant performance in the process of soil remediation, plants can be inoculated with competent soil microorganisms, specifically arbuscular mycorrhizal fungi (AMF) (Ferrol et al. 2016).

AMF are ubiquitous in terrestrial environments and form intimate relationships with the majority of vascular plants (Smith and Read 2008), reflecting the strategic importance of plant-AMF interactions for environmental adaptation (Wang and Qiu 2006). Several studies reported that certain arbuscular mycorrhizal (AM) fungal species facilitate HM transport to aboveground plant organs (phytoextraction) (Weissenhorn et al. 1995; Fiqri et al. 2016; Singh et al. 2019). Other studies, however, revealed that AM fungal species inhibit HM translocation by various means (Shabani et al. 2016; Chamba et al. 2017; Salazar et al. 2018). These include the binding of HM in AM fungal hyphae and glomalin, or sequestering HM in fungal structures (arbuscules, vesicles, vacuoles). With this, the transport of HM to the plant stem is prevented (phytostablization) (Garg and Singh 2018; Motaharpoor et al. 2019). The extent of HM uptake and the subsequent translocation *in planta* further depends on the HM concentration in soils, even in the presence of the same AM fungal species (Huang et al. 2017).

Hg exists in three forms, i.e., elemental Hg, inorganic Hg (Hg^1+^, Hg^2+^) and organic Hg (Beckers and Rinklebe 2017). In soils, the predominant form is Hg^2+^ (Beckers and Rinklebe 2017; Kumari et al. 2020). Uptake of Hg^2+^ via roots is facilitated by an active process (Esteban et al. 2008; Wang et al. 2014; Ma et al. 2021), yet no precise membrane transporters involved in root Hg^2+^ have been identified. It is generally accepted that HM enter roots via nutrient transporters (Manoj et al. 2020) because of the structural similarity of HM with essential nutrients. For example, arsenate (AsO_4_^3−^) is taken up by the same transporters as phosphate (PO_4_^3−^) (Meharg and Hartley-Whitaker 2002) and arsenite shares transporters with silicic acid (Ma et al. 2008). Cadmium (Cd^2+^) uptake by plants is facilitated via zinc (Zn) protein carriers (Kaur and Garg 2018). Such pertinent information is lacking for Hg, however, despite that Hg^2+^, like Cd^2+^, shares the same outer electronic configuration as Zn^2+^ (Jensen 2003). Competition between Hg^2+^ and Zn^2+^ was observed in bacteria upon addition of Zn to a Hg-containing growth solution. These findings imply that Hg and Zn share affinity for the same transporters (Schaefer et al. 2014; Szczuka et al. 2015). Zn is an essential micronutrient for plants, playing vital roles in cellular and physiological functions (Fariduddin et al. 2022). Zn has a low mobility in soil solution, whereby its uptake is diffusion-limited (Lehmann et al. 2014). AMF have been recognized to play a key role in facilitating Zn tissue concentration (Ruytinx et al. 2020). These results highlight the importance of investigating the interplay between Zn and Hg uptake in relation to mycorrhizal colonization, which is fundamental to understand the mechanisms of Hg uptake and accumulation in mycorrhizal plants. Such advanced knowledge would help to optimize AMF-supported plant performance in phytoremediation.

Among the involved transporters of Zn into the cytoplasm, the zinc-iron-regulated transporter (*ZRT-IRT*) family, called *ZIP*, mostly has been studied. In *Medicago truncatula*, four *ZIP* transporters—*ZIP1*, *ZIP2*, *ZIP5*, and *ZIP6* facilitating the transport of Zn^2+^—have been verified in yeast complementation assays (Burleigh et al. 2003; Stephens et al. 2011). Recent studies showed that only two of them are influenced by mycorrhizal colonization, yet under contrasting Zn conditions. Namely, *ZIP6* was up-regulated at deficient and sufficient soil Zn concentrations, while *ZIP2* was up-regulated at toxic Zn concentrations (Watts-Williams et al. 2017).

In this study, we used the model legume *Medicago truncatula* inoculated with the AM fungus *Rhizophagus irregularis* to gain fundamental insights into the underlying mechanisms of Hg uptake and accumulation by mycorrhizal plants. The aims of this study were to (1) examine the effect of *R. irregularis* on biomass and Hg accumulation of *M. truncatula* under Hg exposure; (2) determine the translocation strategies of Hg in roots, stems and leaves of *M*. *truncatula* associated with *R. irregularis*; and (3) investigate the effect of *R. irregularis* on Zn nutrient uptake and Zn transporters (*ZIP2*, *ZIP6*) under Hg exposure.

## Materials and methods

### Preparation of biological materials

*Medicago truncatula* cv. Jemalong A17 seeds were scarified in 90% sulfuric acid for 7.5 min. Seeds were washed eight times with cold water to remove the acid, followed by 90 s surface sterilization in 3% active chlorine solution (sodium hypochlorite solution) (Garcia et al. 2006). The chlorine solution was removed by rinsing of the seeds in sterile water for 5–6 times. Sterilized seeds were soaked in sterile water overnight under darkness at room temperature (20 °C). Then, the seeds were stratified in water-agar plates (0.8% (*w*/*v*)) for 24 h at 4 °C and germinated at 20 °C for 2 days in the dark. After germination, seeds were exposed to light for 2 days to initiate chlorophyll development. The AM fungus *Rhizophagus irregularis* (QS81) was provided by INOQ GmbH (Schnega, Germany). The inoculum of *R. irregularis* was prepared from arbuscular mycorrhizal root fragments of *Trifolium partensis* produced in sand/vermiculite 35/65 v/v in year 2019. The product was filtered through a 425 µm mesh (grade II) and finally contained 100 million propagules kg^−1^ powder (as vesicles and spores according to the manufacturer). Prior to use, the AM fungal propagules were stored in an air-dried, well-ventilated, and dark environment.

### Experiment design and conditions

Sand quartz was twice autoclaved (121 °C/2 h) over a 2-day interval. For the AM fungus inoculated treatment (AM), 50 ml (80 g) sterilized sand were mixed with 25 mg (a ratio of 0.5 g L^−1^ substrate) Osmocote Exact Mini 3–4 months (NPK 15:3.9:9.1 + 1.2 Mg + trace elements, ICL Specialty Fertilizers, UK) (Senovilla et al. 2020), and 160 mg (a ratio of 3.2 g L^−1^ substrate) AM fungus inoculum (Mercy et al. 2017) was added to each plastic pot (5 cm of height, 4 cm of width and with 3 holes in the bottom). For the negative control without inoculum (NM), the same amount of sand mixed with 25 mg Osmocote Exact Mini was filled into each plastic pot. Then, one seedling (5 days) was transferred to each pot. Plants were grown in the greenhouse from 19 June to 17 August, 2021. Plants were maintained at an average temperature of 29.4 °C and an average relative humidity of 51% under natural light conditions (Phytotechnikum research station, University of Hohenheim, Stuttgart, Germany). Plants were watered daily with 5-ml tap water which did not drain from the pots. After 3 weeks, when plants showed vigorous growth, 5 ml HgCl_2_ solution at concentrations of 25 µg g^−1^ or 50 µg g^−1^ were added to each Hg treatment pot once, respectively. The reference pots without Hg application were treated with sterile water in equivalent volume. The Hg treatment was assigned to Hg0, Hg25, and Hg50. The experiment was a 2 × 3 complete factorial design, comprising 5 replications per treatment arranged in a randomized block design. The experiment was performed for 5 weeks after Hg additions until destructive harvest.

### Plant harvest

At the end of experiment (8 weeks), the roots were quickly washed with tap water and separated into 3 parts. One sub-sample (100 mg) of fresh root was immediately flash frozen in liquid nitrogen and stored at −80 °C for RNA isolation. The second sub-sample also was stored at −80 °C for AM root colonization observation. The remaining roots as well as stem and leaf biomass were dried at room temperature until weight stability to determine plant biomass weight.

The tolerance index (TI) was calculated (Eq. 1) to reflect the ability of the host plant to grow in the presence of different Hg concentration (Huang et al. 2017). There, *mt* is the total dry biomass of the plant growing in each pot; *mc* denotes the average total dry biomass of the plants growing in pots without Hg under NM and AM treatment, separately.

1 $$TI=\frac{mt}{mc}$$

### Determination of Hg and Zn

Hg and Zn concentrations were determined separately for roots, stems, and leaves. Each air-dried sample was milled with a centrifugal mill (Retsch GmbH, Haan, Germany) equipped with a titanium rotor and a ring sieve. Samples (0.2 g) were moistened with 1 ml of deionized H_2_O and digested in 2.5 ml 69% HNO_3_ in an UltraCLAVE III microwave heated digestion unit (MLS-MWS GmbH, Leutkirch, Germany). After digestion, the solutions were filled up to 10 ml with deionized H_2_O. Hg concentration was analyzed via a NexION 300 × inductively coupled plasma mass spectrometry (ICP-MS) (PerkinElmer LAS GmbH, Rodgau, Germany), and Zn concentration was analyzed by an Agilent5100 inductively coupled plasma optical emission spectrometry (ICP-OES) (Agilent Technologies GmbH, Waldbronn, Germany) (Core Facility, University of Hohenheim, Stuttgart, Germany).

### Mycorrhizal colonization

Frozen roots stored for AMF observation were cut into 1 cm segments and cleared using 10% NaOH in a 70 °C water bath for 45 min and then soaked in 1% HCl for 1 min at room temperature. The roots were stained in 2% PARKER QUINK blue ink (Yon et al. 2015) in a 70 °C water bath for 30 min. The roots were rinsed with tap water until the water appeared transparent and then were stored in a lactoglycerol solution (v:v:v–1:1:1-latic acid:glycerol:H_2_O). Thirty fragments were randomly selected, placed on a slide, and checked under a light microscope for intraradical AM structures (Trouvelot et al. 1986).

### RNA isolation and quantitative RT-PCR

Total RNA was isolated from 100 mg root sub-samples (RNeasy® plant Mini Kit, QIAGEN GmbH, Germany). RNA integrity was checked by gel electrophoresis, following quantification of RNA by Nanodrop 2100 (Thermo Fisher Scientific). The cDNA was synthesized from 500 ng of RNA using the QIAGEN QuantiTect® Reverse Transcription Kit (QIAGEN GmbH) including a genomic DNA elimination step. Quantification of the expression of *ZIP2*, *ZIP6,* and *Ri-tubulin* genes was conducted with 1 µl of 10 × diluted cDNA in a 20 µl reaction using gene-specific primers and SYBR® green PCR Master Mix in the StepOnePlus™ Real-Time PCR system (Applied Biosystems, USA). The RT-PCR settings were 94 °C for 5 min, then 94 °C for 30 s, 60 °C for 30 s and 72 °C for 30 s for 40 cycles, followed by generation of a dissociation curve. *MtASPP* and *β-actin* were selected as housekeeping genes using NormFinder (Mestdagh et al. 2009). Gene expressions were normalized to the geometric mean of the two selected housekeeping genes (Vandesompele et al. 2002). Table S1 displays the sequences of the forward and reverse primers used in this study.

### Statistical analysis

All statistical analyses were performed in R version 4.0.3. Homogeneity and normality of data were checked with Levene’s test and Shapiro–Wilk test, respectively. Data, which did not meet the criteria, were Box-Cox transformed (Box and Cox 1964). Values presented in figures are non-normalized data. Response variable data were subjected to two-way analysis of variance (ANOVA), with the factors “Hg treatment” and “AM inoculation.” Following two-factor ANOVA, the means of AM fungus inoculation (AM) and non-inoculated control (NM) were compared separately at each Hg level (Hg0, Hg25, Hg50) using Student’s *t*-test (5%)*.* For response variables, for which no AM fungus data could be recorded (i.e., mycorrhizal colonization and *α-tubulin* expression of *R. irregularis* in NM treatments), one-way ANOVA along with Tukey’s honestly significant difference (HSD) was used considering Hg treatment as the only factor. A correlation matrix among Hg concertation and Zn concentration in each plant part, as well as *ZIP* transporter gene expression, was calculated for AM and NM treatments, respectively. Spearman’s method was used, and *P* value was adjusted with a Bonferroni correction.

## Results

### Plant Hg tolerance and mycorrhizal colonization

**Fig. 1:**
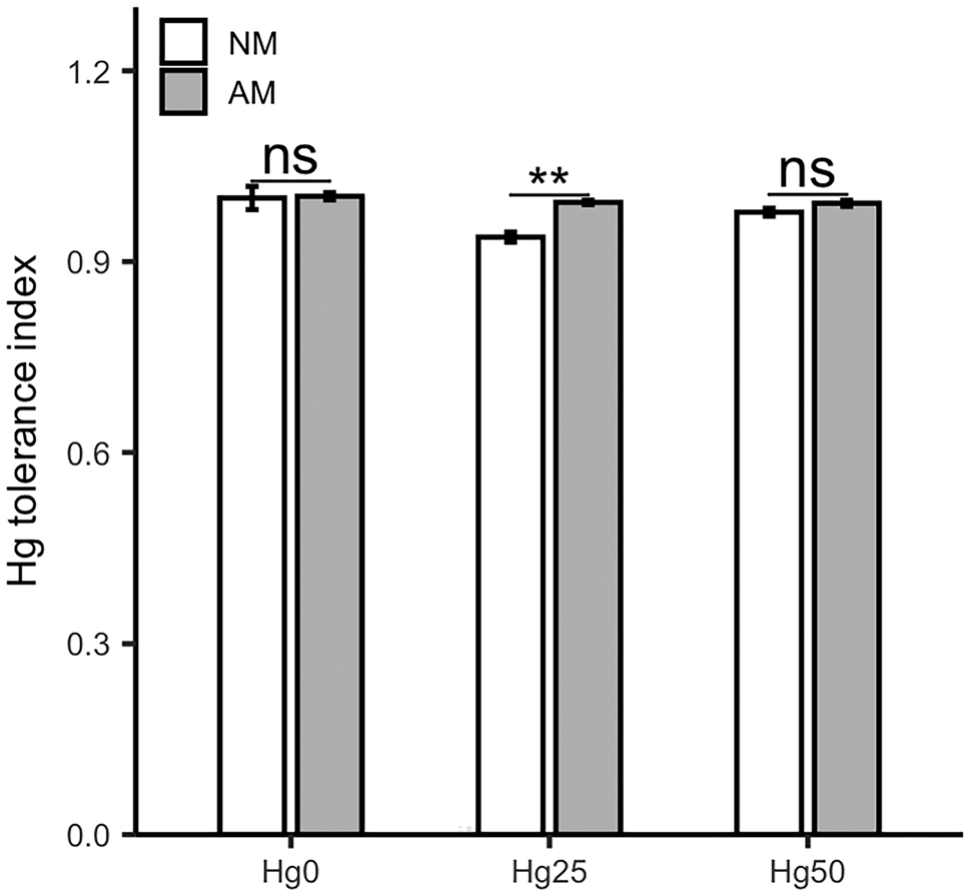
Plant mercury (Hg) tolerance index. Values present mean ± SE (*n* = 5). Asterisks (*) denote a significant mean difference between NM and AM treatments, as measured by the *t-test* (5%). Significance levels: **P < 0.01; ns, not significant (*P* > 0.05)

Overall, inoculation with *Rhizophagus irregularis* (AM plants) conferred higher Hg tolerance to *Medicago truncatula* compared to non-inoculated controls (NM plants) (*P* < 0.001) (Table 1). This effect was significant for Hg25 (*P* < 0.01) (Fig. 1). The results of dry biomass in each part (leaves, stems, and roots) and total biomass are shown in Table S2. Leaf necrosis in NM plants under Hg50 treatment was observed.

**Table 1:** Summary of ANOVA results for all response variables. Factors in the analysis were Hg treatment and AM inoculation (*Rhizophagus irregularis*). Both the main effects and interaction term are indicated where relevant. Significance levels: *P < 0.05; **P < 0.01; ***P < 0.001; ns, not significant (*P* > 0.05)

	Hg treatment	AM inoculation	Hg treatment x AM inoculation
Mycorrhizal colonization (%)	ns		
*Ri α-tubulin* expression	ns		
TI	ns	***	ns
Leaf Hg concentration	ns	***	ns
Stem Hg concentration	***	ns	***
Root Hg concentration	***	ns	***
Leaf Hg content	ns	***	ns
Stem Hg content	ns	ns	**
Root Hg content	***	ns	ns
Leaf Zn concentration	ns	**	*
Stem Zn concentration	ns	***	ns
Root Zn concentration	ns	**	ns
Leaf Zn content	ns	ns	ns
Stem Zn content	ns	*	ns
Root Zn content	ns	ns	ns
*ZIP2* expression	ns	**	ns
*ZIP6* expression	ns	***	ns

**Fig. 2:**
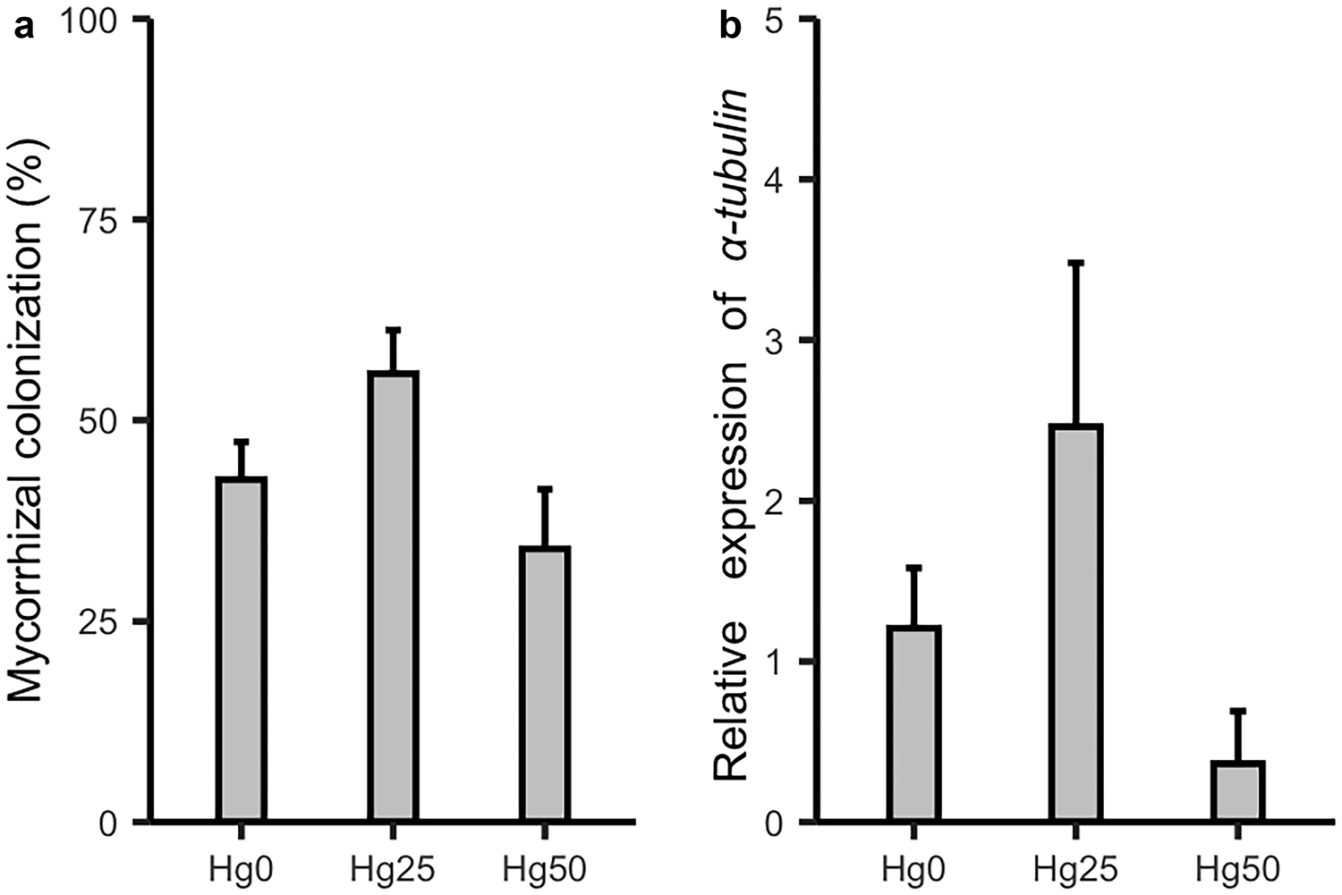
Percentage of mycorrhizal colonization **a **and relative expression of AM fungal biomass marker gene *α-tubulin ***b **in roots of *Medicago truncatula* plants inoculated with *Rhizophagus irregularis* and grown at different Hg concentrations. Values present mean ± SE (*n* = 5). There were no significant effects of Hg treatment on both mycorrhizal colonization and relative expression of *α-tubulin* (see Table 1 for detailed ANOVA analysis)

Under Hg0, Hg25, and Hg50, frequencies of mycorrhizal colonization of 42.7%, 55.8%, and 34%, respectively, were determined but did not differ significantly (*P* > 0.05) (Fig. 2a, Table 1). Pictures of colonization are shown in Fig S1. The expression of the *α-tubulin* gene in mycorrhizal roots of AM plants followed the same trend (Fig. 2b). Non-inoculated roots (NM plants) were free of mycorrhizal colonization, as confirmed by lack of *α-tubulin* gene expression in their roots.

#### Concentration and accumulation of Hg

**Fig. 3:**
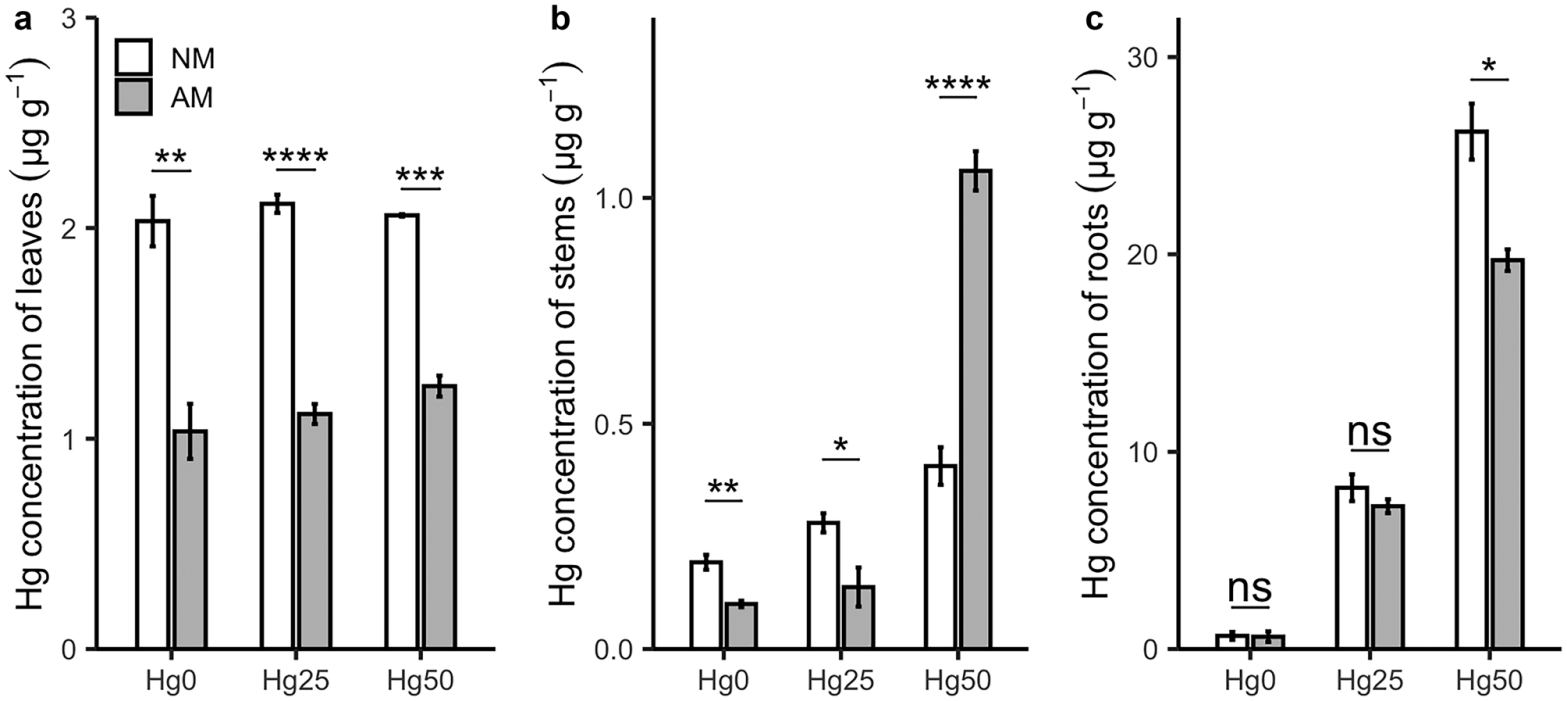
Mercury (Hg) concentration in leaves **a**, stems **b**, and roots **c **of non-mycorrhizal (NM) and mycorrhizal (AM) plants exposed to three different Hg levels (Hg0, Hg25, Hg50). Values present mean ± SE (*n* = 5). Asterisks (*) denote a significant mean difference between NM and AM treatments, as measured by the *t-test* (5%). Significance levels: *P < 0.05; **P < 0.01; ***P < 0.001; ****P < 0.0001; ns, not significant (*P* > 0.05)

**Fig. 4:**
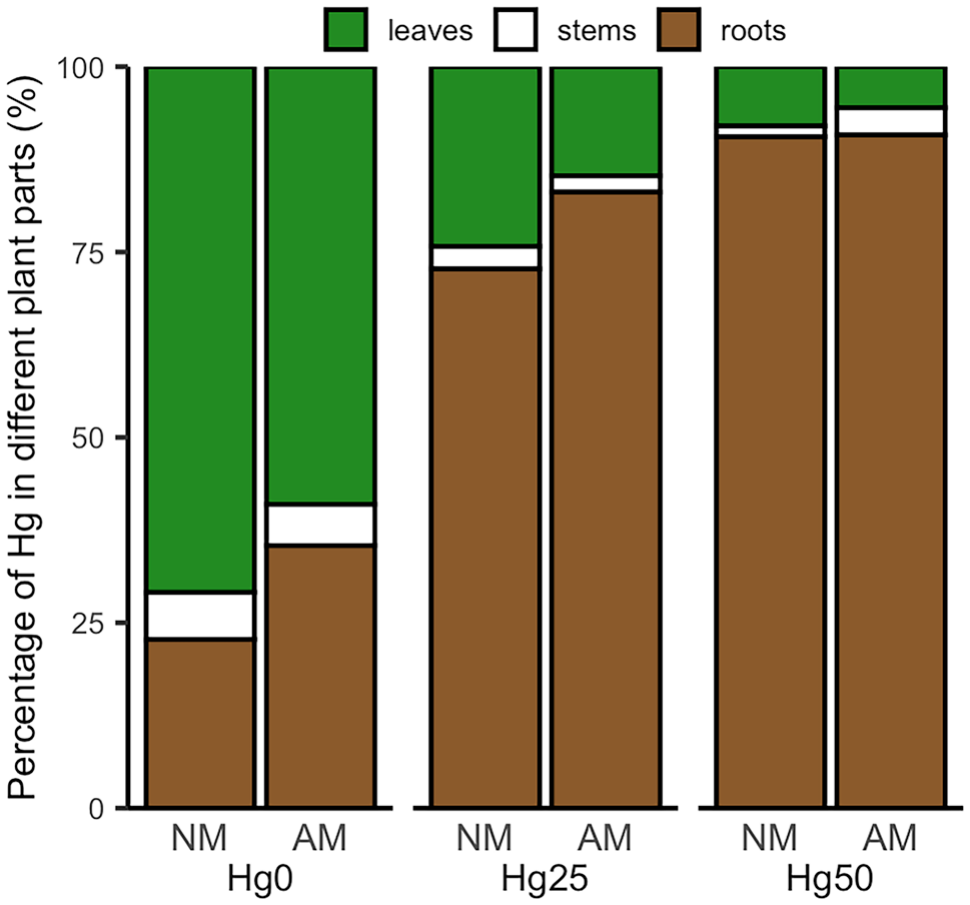
Percentage mercury (Hg) content (= concentration × dry weight) in different plant parts of non-mycorrhizal (NM) and mycorrhizal (AM) plants exposed to three different Hg levels (Hg0, Hg25, Hg50)

Figure 3 shows the Hg concentration in different plant parts (leaves, stems, roots). AM inoculation reduced the Hg concentration in leaves at all Hg concentrations compared to NM plants (*P* < 0.001) (Table 1, Fig. 3a). Hg concentrations in leaves at different Hg treatments were similar (Fig. 3a). There was no Hg contamination detected in the control substrate (Hg0) after the experiment (Fig. S2). Therefore, Hg accumulation in leaves likely results from absorbance of atmospheric Hg, because all plants were grown in the same compartment in the greenhouse. There was a significant interaction between Hg treatment and AM fungus inoculation for Hg concentration of stems (*P* < 0.001) (Table 1). The Hg stem concentration under Hg25 was lower in AM plants than in NM plants (*P* < 0.05) (Fig. 3b). Conversely, under Hg50, the Hg stem concentration was higher in AM plants than in NM plants (*P* < 0.0001) (Fig. 3b). For roots, there was a significant interaction between Hg treatment and AM fungus inoculation (*P* < 0.001) (Table 1), which was most prominent under Hg50 (*P* < 0.05) (Fig. 3c). Concerning Hg contents in different plant parts, AM fungus inoculation significantly reduced the Hg content in leaves compared to NM plants (*P* < 0.001) (Table 1). There also was a significant interaction between Hg treatment and AM inoculation for Hg stem content (*P* < 0.01) (Table 1). For Hg root content, only Hg treatment had a significant main effect (*P* < 0.001) (Table 1). Figure 4 shows the percentage of Hg content in each plant part, substantiating the root as prominent plant tissue for Hg accumulation under Hg treatment (Fig. 4).

### Zn concentration and Zn transporter gene expression

**Fig. 5:**
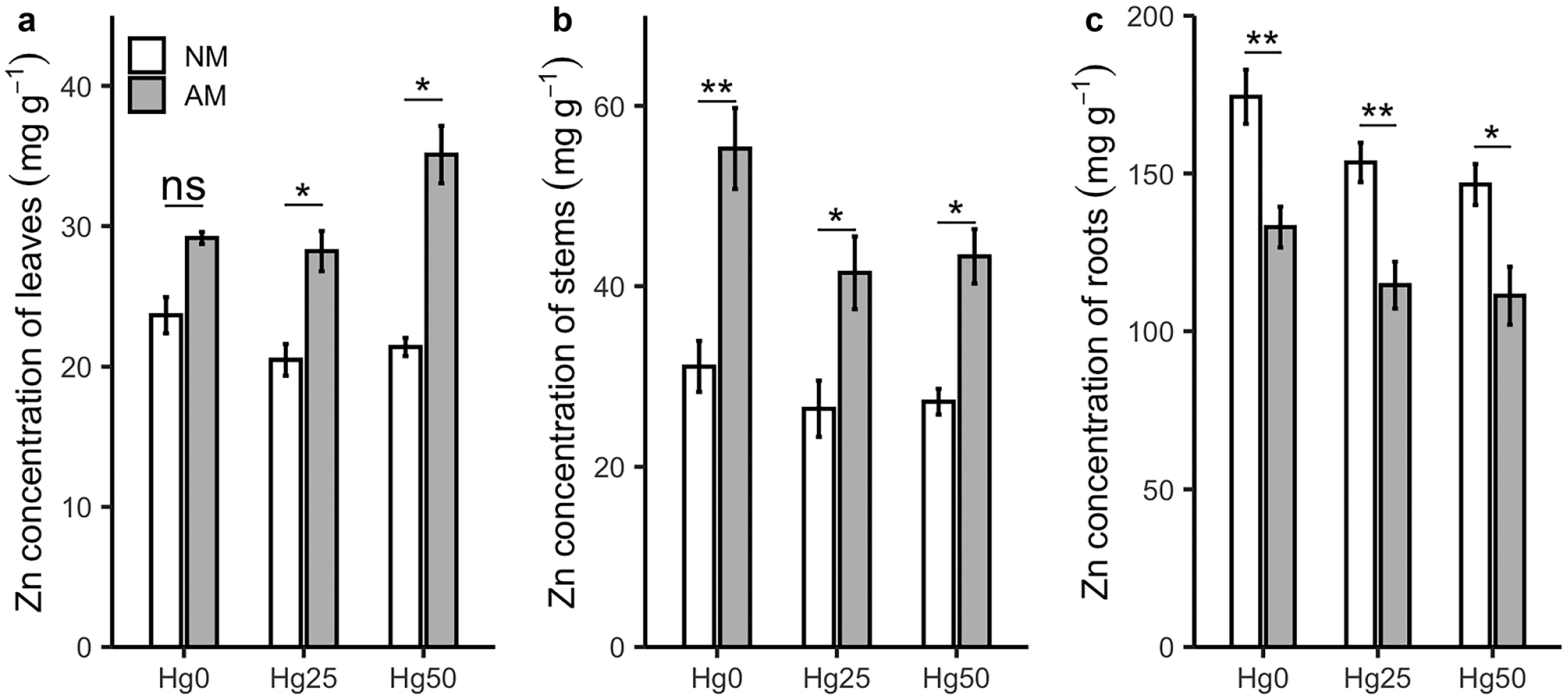
Zinc (Zn) concentration in leaves **a**, stems **b**, and roots **c **of non-mycorrhizal (NM) and mycorrhizal (AM) plants exposed to three different mercury (Hg) levels (Hg0, Hg25, Hg50). Values present mean ± SE (*n* = 5). Asterisks (*) denote a significant mean difference between NM and AM treatments, as measured by the *t*-test (5%). Significance levels: *P < 0.05; **P < 0.01; ***P < 0.001; ns, not significant (*P* > 0.05)

**Fig. 6:**
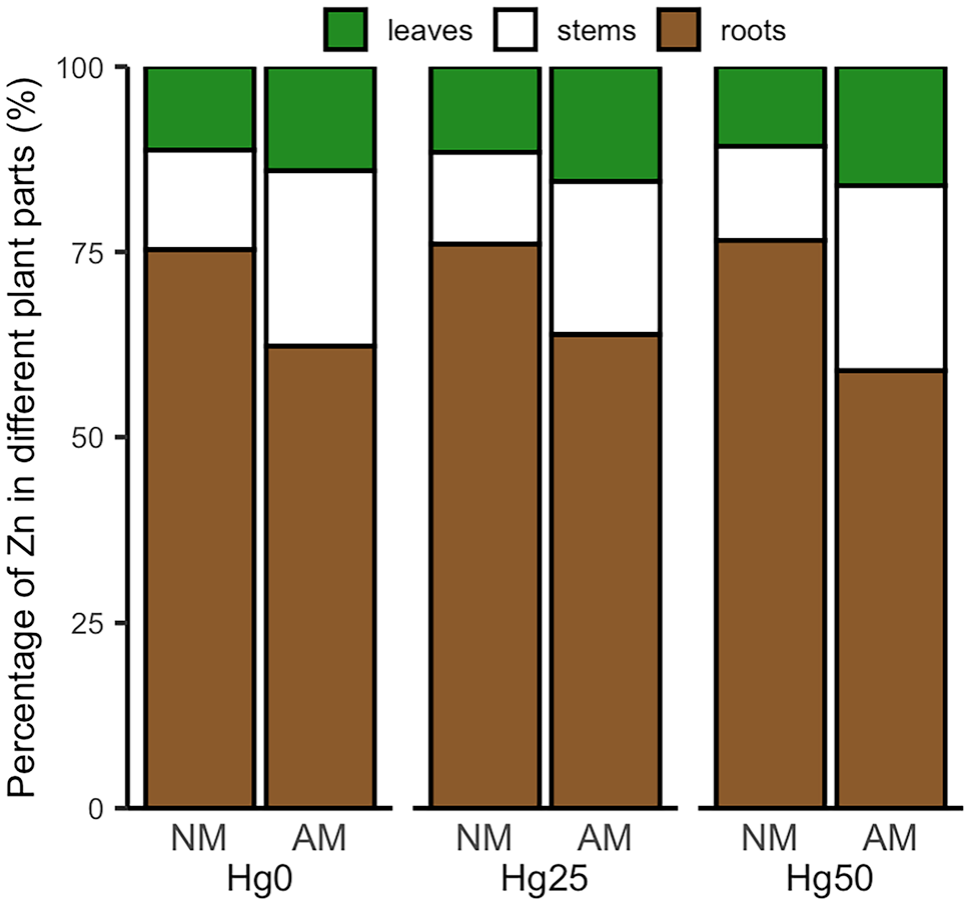
Percentage zinc (Zn) content (= concentration × dry weight) in different plant parts of non-mycorrhizal (NM and mycorrhizal (AM) plants exposed to three different mercury (Hg) levels (Hg0, Hg25, Hg50

**Fig. 7:**
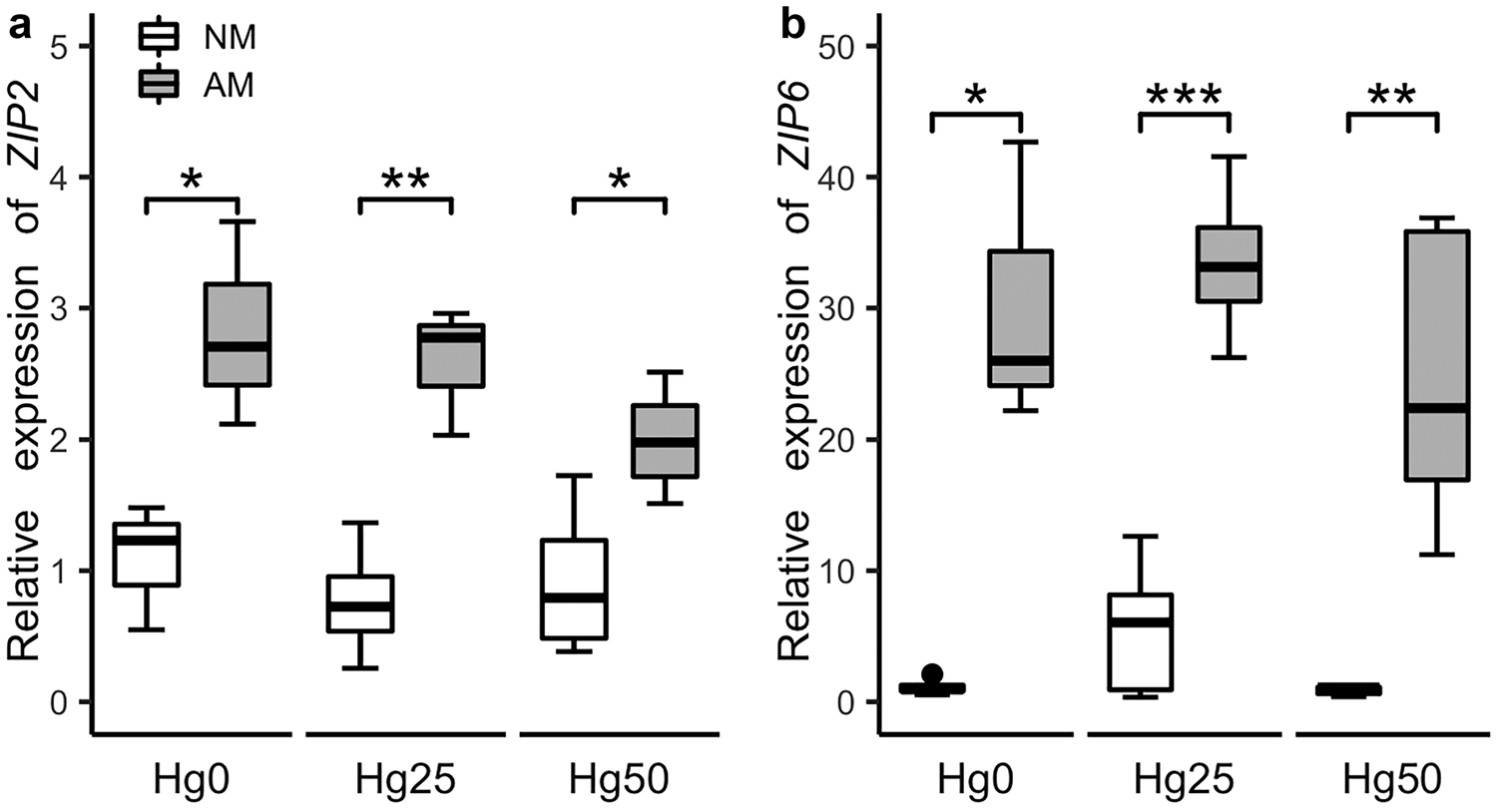
The relative gene expression of *MtZIP2 ***a **and *MtZIP6 ***b **in non-mycorrhizal (NM) and mycorrhizal (AM) plants exposed to three different mercury (Hg) levels (Hg0, Hg25, Hg50). Values present mean ± SE (*n* = 5). Asterisks (*) denote a significant mean difference between NM and AM treatments, as measured by the *t*-test (5%). Significance levels: *P < 0.05; **P < 0.01; ***P < 0.001

There was a significant interaction between Hg treatment and AM fungus inoculation on Zn concentration in leaves (*P* < 0.05) (Table 1). Specifically, AM plants showed higher Zn concentrations than NM plants under Hg25 and Hg50 (*P* < 0.05) (Fig. 5a). AM fungus inoculation increased Zn concentrations in stems compared to NM plants (*P* < 0.001) (Table 1, Fig. 5b). In contrast, AM plants showed lower Zn concentrations in roots than NM plants (*P* < 0.01) (Table 1; Fig. 5c). However, considering Zn content in different plant parts, AM inoculation only had a significant effect on Zn content in stems (*P* < 0.05) (Table 1), by increasing the percentage of Zn content in stems when inoculated with AM fungus (Fig. 6). AM fungal inoculation significantly upregulated the expression of *ZIP2* (*P* < 0.01) and *ZIP6* (*P* < 0.001) genes, irrespective of Hg treatment (Table 1, Fig. 7).

### Correlations between Hg and Zn concentrations in roots

Correlation matrices among measured parameters were calculated for NM and AM treatments separately (Table S3 and Table S4). Hg concentration in stems was negatively correlated with Zn concentration in roots of the NM plants (*r* =  −0.74; *P* < 0.05), and Hg concentration in roots was negatively correlated with Zn concentration in stems under AM treatment (*r* =  −0.70; *P* < 0.05). Interestingly, Hg concentration in roots was negatively correlated with Zn concentration in roots for both NM and AM treatments (*r* =  −0.71; *r* =  −0.70, respectively; *P* < 0.05).

## Discussion

### Effects of Rhizophagus irregularis on Hg tolerance index and Hg partitioning

Numerous studies have indicated that AMF inoculation could alleviate HM stress of plants (Garg and Singh 2018; Motaharpoor et al. 2019; Molina et al. 2020). In line with this, our results showed a positive effect of *Rhizophagus irregularis* on Hg tolerance of *Medicago truncatula*, suggesting the potential of using AM fungi in Hg phytoremediation. Furthermore, our results showed the effects of *R. irregularis* on Hg partitioning in each plant part (leaves, stems and roots). There was a significant reduction of Hg concentration and content in leaves of AM plants compared to NM plants, indicating that the strain of *R. irregularis* was able to protect leaves from Hg uptake. Assad et al. (2016) demonstrated that Hg uptake by leaves is exclusively caused through the atmospheric pathway. This also was indicated in our study in which leaf Hg concentration was similar under different Hg concentration treatments. Thus, the reduction of Hg concentration in leaves might be attributed to the regulation of stomatal closure by *R. irregularis*. This possibility deserves further investigation. Wang et al. (2017) showed that stomatal closure is part of the protective strategy initiated by *R. irregularis* under exposure to cadmium.

Additionally, a significant interaction between Hg treatment and AM inoculation on Hg stem concentration and content was detected in our study, indicating that the effect of *R. irregularis* on Hg translocation to stems was dependent on Hg concentration in the substrate. Prior work showed that maize associated with *Funneliformis mosseae* (formerly *Glomus mosseae*) did not influence Hg shoot concentration compared to non-inoculated plants exposed to Hg concentrations of 2 and 4 mg kg^−1^ (Yu et al. 2010). Recent studies have demonstrated, however, that *Glomus* sp. associated with maize facilitated Hg uptake and its translocation from roots to shoots at a Hg concentration of 50 mg kg^−1^ (Kodre et al. 2017; Debeljak et al. 2018). Together, these results indicate that the role of AM fungal species in Hg translocation in plants is likely dependent on the Hg concentration in the substrate, as was corroborated by our study.

The observed increase of Hg concentrations in roots with increasing Hg levels was consistent with other reports. For example, Hg concentration in both lupin and maize roots showed a hyperbolic pattern with increasing Hg additions in the growth solution (Esteban et al. 2008; Yu et al. 2010). Furthermore, AM inoculation reduced Hg concentration at Hg50, indicating a protective role of *R. irregularis* under high Hg concentration. This reduction was generally attributed to the role of AM fungal structures, e.g., binding with hyphae and sequestration into vacuoles. However, this may not be the case in the present study because such a reduction was not observed under Hg25. There is no difference in Hg root concentrations between NM and AM treatments under Hg25, indicating the capacity of root Hg accumulation of *M*. *truncatula*. Within this capacity, plants can detoxify Hg by themselves (Kumar et al. 2017), but plants may need support from microbes (in this case *R. irregularis*) at high concentrations, as it was shown under Hg50. Accordingly, further work will be necessary to precisely investigate the functions of the same AM fungal species on different plant species exposed to different Hg concentrations.

### Effects of Rhizophagus irregularis on Zn under Hg exposure

Generally, AM inoculation exerts a positive effect on the transport of elemental nutrients (Smith and Read 2008). In our study, AM inoculation increased Zn concentration in aboveground plant parts exposed to Hg, a finding in line with other studies (Debeljak et al. 2018; Saboor et al. 2021). Moreover, AM fungus inoculation upregulated the expression of Zn transporter genes (*ZIP2*, *ZIP6*), independent of Hg levels in the substrate. This indicates that Hg uptake by roots might not have been related to these two transporters in our study. This also was indicated by the absence of correlations between Hg root concertation and the two assayed *ZIP* transporter genes. Nevertheless, negative relationships between Hg and Zn concentrations in the roots of both AM and NM plants were found, implying potential competition between both elements for the same transporters. Our results suggest that Hg did not impair the positive regulation of Zn nutrient and its transporter genes by *R. irregularis*, which may have contributed to Hg tolerance because Zn plays a vital role in the reduction of the oxidative stress caused by Hg (Calgaroto et al. 2011). In order to fully understand the relationship between both Zn and Hg, further experiments with different Zn concentrations in the substrate and studies on other Zn-related transporter genes are needed.

## Conclusions

Our results showed that the regulatory role of *R. irregularis* in Hg accumulation and translocation from roots to stems in *Medicago truncatula* is dependent on the concentration of Hg in the substrate. Additionally, a positive effect of *R. irregularis* on Hg tolerance of *M. truncatula* along with improvement of Zn nutrient status and upregulation of Zn transporter genes (*ZIP2*, *ZIP6*) was found.

## Supplementary Information

Below is the link to the electronic supplementary material.

Supplementary file1 (PDF 1460 KB)

Supplementary file2 (XLSX 16 KB)

## Data Availability

All data generated or analyzed during this study are included in this published article and its supplementary information files.
