# Supplementary material for: Rhizophagus irregularis improves Hg tolerance of Medicago truncatula by upregulating the Zn transporter genes ZIP2 and ZIP6
Source: Mycorrhiza. 2023 Jan 10;33(1-2):23–32. doi: 10.1007/s00572-022-01100-6 (PMC9938064; doi:10.1007/s00572-022-01100-6)
Supplement: Supplementary file 1 — Supplementary file1 (PDF 1460 KB) [file 572_2022_1100_MOESM1_ESM.pdf]

Online Resource 1

Rhizophagus irregularis* improves Hg tolerance of *Medicago truncatula* by upregulating the Zn transporter genes *ZIP2* and *ZIP6

Yaqin Guo[1], Nadine Sommer[2], Konrad Martin[1], Frank Rasche[1*]

[1]Institute of Agricultural Sciences in the Tropics (Hans-Ruthenberg-Institute), Department of Agronomy in the Tropics and Subtropics, University of Hohenheim, 70593, Stuttgart, Germany

[2]Institute of Crop Science, Department of Crop Physiology of Specialty Crops, University of Hohenheim, 70593, Stuttgart, Germany

***Corresponding author:** Dr. Frank Rasche

E-mail: frank.rasche@uni-hohenheim.de

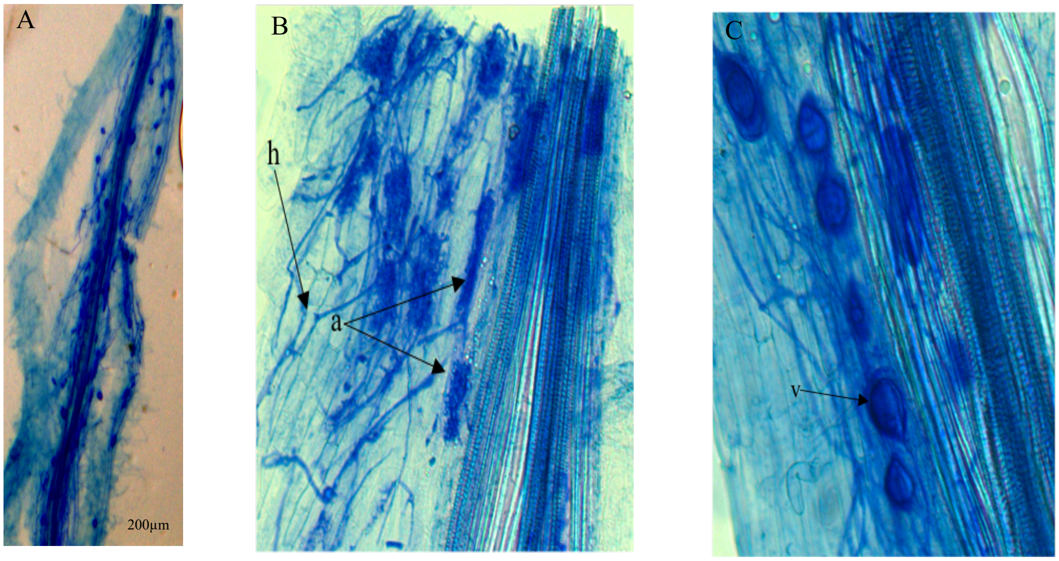

Fig S1. Arbuscular mycorrhiza colonization of plant roots. (A) *Medicago truncatula* root colonized with *Rhizophagus irregularis*; (B) arbuscules (a) and hyphae (h); (C) Vesicles (v). Fungal structures were stained with ink and vinegar

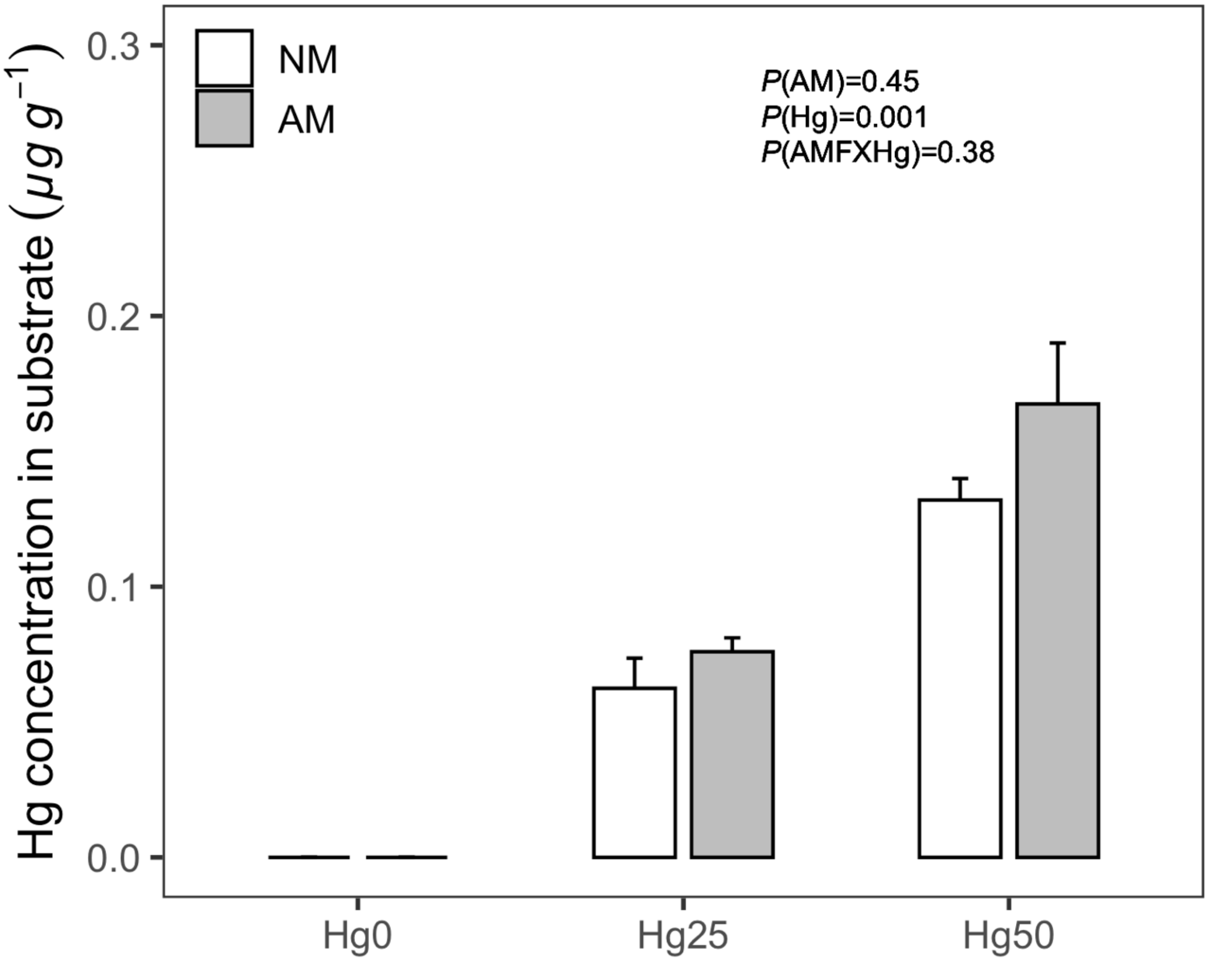

Fig S2. Hg concentration in substrate in both non-mycorrhizal (NM) and arbuscular mycorrhizal (AM) treatment with three different Hg levels (0, 25 and 50 μg g^{-1}) after experiments. The values present mean ± SE (n=5). Under Hg0, there was no detection of Hg, indicating that there is no Hg contamination in the control substrate. Results of two-way ANOVA were annotated in the figure. There were no significant differences between NM and AM treatment means
